# Quality of Meat and Fat from Immunocastrated Boars and Uncastrated Boars Slaughtered at Different Body Weights

**DOI:** 10.3390/ani15233374

**Published:** 2025-11-21

**Authors:** Anna Zalewska, Marcin Sońta, Justyna Więcek, Anna Rekiel, Piotr Cybulski, Iwona Wojtasik-Kalinowska, Andrzej Półtorak, Kamila Puppel, Martyna Batorska

**Affiliations:** 1 Department of Animal Breeding and Nutrition, Institute of Animal Sciences, Warsaw University of Life Sciences, Ciszewskiego 8 Street, 02-786 Warsaw, Poland; justyna_wiecek@sggw.edu.pl (J.W.); anna_rekiel@sggw.edu.pl (A.R.); kamila_puppel@sggw.edu.pl (K.P.); martyna_batorska@sggw.edu.pl (M.B.); 2 Goodvalley Agro S.A., Dworcowa 25 Street, 77-320 Przechlewo, Poland; piotr.cybulski@goodvalley.com; 3 Department of Technique and Food Product Development, Institute of Human Nutrition Sciences, Warsaw University of Life Sciences, Nowoursynowska 159c Street, 02-776 Warsaw, Poland; iwona_wojtasik-kalinowska@sggw.edu.pl (I.W.-K.); andrzej_poltorak@sggw.edu.pl (A.P.)

**Keywords:** pigs, immunocastration, fattening of uncastrated boars, meat quality, fat quality, boar taint

## Abstract

This study involved 30 male pigs (DanBred × Duroc), which were divided into three groups of 10 animals each. Control group (C)—immunologically castrated boars with a slaughter weight of 120 kg; and experimental groups: E1—uncastrated boars with a slaughter weight of 120 kg, and E2—uncastrated boars with a slaughter weight of 105 kg. Animals from all groups were fed a complete feed mixture in a liquid form three times a day. After slaughter, their meat and backfat were analysed for the physicochemical parameters and for the contents of indole, skatole, androstenol, and androsterone. A higher protein content was determined in the meat of boars from group E1 (23.48%) compared to those from groups C (22.87%) and E2 (22.99%) (*p* ≤ 0.01), and a higher content of n-6 PUFAs in the meat of boars from group C (5.21 mg/g of meat) compared to those from group E2 (4.81 mg/g of meat) (*p* ≤ 0.05). Analysis of the chemical composition of backfat showed a lower protein level in the backfat of boars from group C (4.70%) compared to those from group E1 (6.20%) and a higher fat level in the backfat from boars from group C (70.09%) compared to those from groups E1 (65.90%) and E2 (64.75%) (*p* ≤ 0.05). Body weight and immunocastration status were also shown to affect the fatty acid profile. Immunocastration also reduced the content of androstenol and androsterone in meat and fat. A higher content of indole was demonstrated in the meat of boars from group C and in the backfat of those from group E2 compared to the animals from the other groups (*p* ≤ 0.001).

## Simple Summary

The abandonment of surgical castration of boars in pork production creates the need to implement alternative solutions that will eliminate boar taint in meat and fat, while preserving their desired physicochemical properties. This study aimed to analyse the quality of meat and fat from immunocastrated boars and uncastrated boars with slaughter weights of 120 and 105 kg. Its results showed that the slaughter of uncastrated boars with a body weight of 120 kg had a beneficial effect on the protein content of their meat. However, the lack of castration resulted in fat quality deterioration. In turn, the immunocastration reduced the content of hormones responsible for the presence of boar taint in meat and fat.

## 1. Introduction

The use of entire male pigs in pork production can lead to boar taint in meat and fat, an unpleasant odour and flavour in meat and fat caused primarily by the accumulation of androstenone, skatole, and indole in the adipose tissue [1]. The odour is described as urinary or faecal, as well as sweat or manure, and its perception depends on individual consumer sensitivity [2]. The occurrence of boar taint is strongly related to the sexual maturity of males. Androstenone is produced in the Leydig cells of the testicles and acts as a pheromone, signalling readiness for reproduction [3]. Unmetabolised androstenone is stored in the adipose tissue [2]. Skatole, in turn, is a product of the bacterial breakdown of tryptophan in the large intestine. Although it is not a sex hormone, it is closely related to the function of androstenone. This is because sex steroids inhibit the metabolism of skatole, leading to its accumulation in tissues [3,4]. The conventional method of preventing boar taint, i.e., surgical castration, has raised ethical concerns among consumers and is legally restricted is some countries, such as Germany, Belgium, France, Norway, and Sweden [5]. Surgical castration is often performed without anaesthesia up to the seventh day of a piglet’s life. It causes pain and discomfort, which reduce the animals’ welfare and undermine the ethicality of the method [6,7,8]. One practical alternative is immunocastration, which induced the production of specific antibodies against the gonadotropin-releasing hormone (GnRH), leading to the disruption of the hypothalamic–pituitary–gonadal axis and inhibition of testicular function, thereby reducing the production of sex hormones [9]. The effectiveness of immunocastration is achieved by administering the preparation in two doses. The first dose (at approximately 8 weeks of age) is intended to ensure immunological preparation for an immune response after the second dose (4 weeks after the first dose). It results in a temporary, complete loss of testicular function, which in turn inhibits the production of substances in the testicles that determine the unpleasant odour of boar meat [10,11,12]. Immunocastration is considered an effective method, but there have been cases where its effect was unsatisfactory, with approximately 0–3% of animals showing no response to the administration of the preparation [11]. Fattening uncastrated males is another solution to prevent the pain caused by the surgical castration. Despite concerns about the risk of boar taint in meat, it can bring benefits by ensuring higher production profitability. Uncastrated males exhibited a higher feed conversion rate and thus greater body weight gains, and their carcasses contain more lean meat and less fat than that of the castrated males [13,14]. Intensive genetic selection for an increased growth rate, improved feed efficiency, and higher lean meat content may also influence the earlier attainment of sexual maturity in modern pig breeds.

The aim of this study was to compare the quality of meat and backfat across three commercially relevant production scenarios: immunocastrated boars with a slaughter weight of 120 kg, uncastrated males with a slaughter weight of 120 kg, and uncastrated males with a slaughter weight of 105 kg.

## 2. Materials and Methods

In accordance with the Polish law and EU Directive No. 2010/63/EU, the experiment did not require approval from the local ethics committee, as it was conducted in production facilities under standard farming practices. The applied procedures were limited to routine nutritional and veterinary treatments performed on the pig farm. Therefore, this study complied with ethical guidelines, ensuring animal welfare without the need for formal ethical evaluation.

### 2.1. Animals

The animals were selected from a group of 70 boars housed in two pens. The first pen (N = 35) contained animals intended for the control group (C), while the second pen (N = 35) contained animals designated for the experimental groups E1 and E2. Boars from group C were subjected to immunocastration. The first dose of Improvac^®^ (Zoetis, Zaventem, Belgium) was administered at 12 weeks of age, and the second dose was given 4 weeks after the first. At the beginning of the fattening period, the average body weight of pigs across all groups was 28.0 kg. After 73 days of fattening, 10 animals from the second pen, with an average body weight of approximately 105 kg, were selected to form group E2. After a further 13 days (86 days of fattening), animals from the same pen with a slaughter weight of about 120 kg were selected to form group E1, along with 10 animals of similar weight from the first pen (group C). As a result, three groups were established, each consisting of 10 pigs: one control group (C) and two experimental groups (E1 and E2) (Table 1).

### 2.2. Feeding

Feeding was standardised across all groups. In the two-phase fattening period (phase I—26 days for groups C, E1, and E2, phase II—60 days for groups C and E1; 47 days for group E2), a complete feed mixture was administered to the animals in the liquid form three times a day. The compound feed used during the first fattening period consisted of the following ingredients: barley—31.0%, triticale—29.0%, soybean meal—29.0%, wheat bran—3.5%, animal fat—0.5%, and mineral–vitamin premix and amino acids—7.0%. During the second fattening period, the following composition was used: barley—55.0%, soybean meal—34.0%, wheat bran—4.0%, animal fat—0.5%, and mineral–vitamin premix and amino acids—6.5%. At each stage of fattening, the above compound feeds were mixed with corn-cob mix (CCM) at a 50:50 ratio and subsequently liquefied with water. The final feed mash contained 59% water. The pigs had ad libitum access to fresh water.

### 2.3. Slaughter of Animals, Slaughter Indicators

After the fattening period, all animals were slaughtered according to the procedures applied in the slaughterhouse, and meatiness (%) was estimated using an ultrasound device—AutoFom III. The half-carcasses were assigned to quality classes in the EUROP system and, consequently, all of them were classified as class S (average meatiness was 61.1%, 63.2%, and 63.1% for groups C, E1, and E2, respectively).

### 2.4. Meat Analyses

After 24 h of cooling the carcasses at +4 °C, a sample of the longest lumbar muscle (*M. longissimus lumborum*—MLL) weighing approx. 0.5 kg was taken from the right half-carcasses from the head section (N = 30) for quality analyses.

Meat colour was determined using the CIELab system: L* (lightness), a* (green/red ratio), and b* (blue/yellow ratio), with a Chroma Meter CR-400/410 (Konica Minolta, Osaka, Japan) [15]. A slice of meat approximately 2 cm thick was measured at three random points, and the results were averaged.

To determine drip loss, a meat sample (approx. 300 g) was placed in a polyethylene bag and stored at +4 °C for 24 h. The weight of the released liquid (muscle juice) was expressed as a percentage relative to the sample weight.

Cooking loss was determined during heat treatment of minced meat according to the method described by Honikel (1998) [16].

Water-holding capacity (WHC) was assessed according to the methodology described by Grau and Hamm (1953) [17], modified by Pohja and Ninivaara (1957) [18].

The contents of water, protein, fat, and collagen were determined in freshly minced pork samples, using a FoodScanTM Lab meat analyser (Foos Analytical, Hillerød, Denmark) [19].

Fatty acid profile: meat fat extraction was performed according to the Folch method [20]. Fatty acid methylation was performed according to the ISO 5509 (2000) [21] transesterification method. Individual fatty acids were identified in crude fat using an Agilent 7890A Gas Chromatograph (Agilent, Waldbronn, Germany) according to the methodology described by Puppel et al. (2013) [22]. Each peak was identified using pure methyl ester standards: FAME Mix RM-6, Lot LB 68242; Supelco 37 Comp. FAME Mix, Lot LB 68887; methyl linoleate, Lot 094K1497; CLA Conjugated (9Z, 11E), Lot BCBV3726 (Supelco, Bellefonte, PA, USA). The following fatty acid groups were determined: SFAs—C14:0, C16:0, C18:0; MUFAs—C16:1, C18:1, C20:1, C22:1; n-6 PUFAs—C18:2, C20:4, C22:2; and n-3 PUFAs –C18:3, C22:5, C22:6. The determined contents of individual fatty acids and fatty acid groups allowed for calculating the atherogenic index (AI) and the thrombogenic index (TI) according to Ulbricht and Southgate (1991) [23] using the following formulas.

AI = (4 × C14:0 + C16:0)/(MUFA + PUFA)

TI = (C14:0 + C16:0 + C18:0)/(0.5 × MUFA + 0.5 × n-6 PUFA + 3 × n-3 PUFA + n-3/n-6 PUFA)

### 2.5. Backfat Analyses

After cooling the carcasses for 24 h at +4 °C, a sample of backfat was taken from the right half-carcass above the neck for laboratory analyses.

The samples were prepared in advance, i.e., the skin was removed and any muscle tissue fragments were cleaned off. The colour of the backfat was measured using a Chroma Meter CR-400/410—Konica Minolta (Konica Minolta, Osaka, Japan). The colour parameters were determined in the CIELab system: L*—lightness, a*—green/red component, and b*—blue/yellow component [15].

The protein content was determined using the Kjeldahl method, while the fat content was analysed by the Soxhlet method.

The fatty acid profile was determined using the same method as for the *M. longissimus lumborum* samples.

### 2.6. Analysis of Compounds Responsible for Odour in Meat and Backfat

The contents of indole (2,3-benzopyrrole), skatole (3-methylindole), androstenol (5α-androst-16-en-3α-ol), and androsterone (5α-androstan-3α-ol-17-one) were determined in minced meat and backfat samples using the HS-SPME-GC/MS method, according to SOP M.032, edition of 04.10.2021, valid in the laboratory.

### 2.7. Instrumental Measurement of Volatile Compounds—e-Nose Analysis

Volatile compounds in meat/backfat were obtained using an electronic nose Heracles II (Alpha M.O.S., Toulouse, France). The method enables rapid identification based on retention indices and odour libraries; however, it does not allow for differentiation between chiral isomers. The method was described in the works by Wojtasik-Kalinowska et al. (2017) [24] and Górska-Horczyczak et al. (2017) [25]. The electronic nose is based on ultra-fast gas chromatography with headspace and consists of a detector system with two metal columns of different polarities (nonpolar MXT-5 and slightly polar MXT1701, diameter = 180 µm, length = 10 m) and also two flame ionisation detectors (FIDs). The Kovats indices were determined based on alkane standards (n-butane to n-hexadecane) (Restek GmbH, Bad Homburg, Germany) measured under the same conditions as the samples. Volatile compounds were identified using the AroChemBase database (Alpha MOS Co., Toulouse, France) containing 44,000 compounds and including also a database of sensory descriptors for each single compound. To this end, 3 g of meat and 3 g of backfat were placed in 20 mL headspace vials and capped with a Teflon-faced silicon rubber cap. Then, the vials with the analysed samples were incubated at 55 °C for 900 s under agitation speed (8.33 Hz). Carrying gas (hydrogen) was circulated at a constant flow rate (1 mL min^−1^). The injector temperature was 200 °C, injected volume was 2500 µL, and injection speed was 125 mL s^−1^. The analytes were collected in the trap at 15 °C and then divided and simultaneously transferred into the two columns. A carrying gas was applied at a constant pressure of 80 kPa. The split flow rate was 10 mL min^−1^ at the column heads. The temperature programme in the oven was set as 60 °C for 2 s; 3 °C s^−1^ ramp to 270 °C and kept for 20 s, and FID1/FID2 at 280 °C. The samples were analysed in four replications.

### 2.8. Statistical Analysis

All data were analysed using ANOVA (IBM SPSS Statistics 25, Armonk, NY, USA). Normality was checked by the Shapiro–Wilk test. Tukey’s HSD post hoc test was applied for pairwise comparisons when assumptions were met; otherwise, Kruskal–Wallis with Dunn’s test was used. Different lowercase letters (a, b) indicate significant differences at *p* ≤ 0.05; uppercase (A, B) indicate *p* ≤ 0.01.

The flavour profile was subjected to the principal component analysis (PCA), using AlphaSoft Version 8.0.

## 3. Results

### 3.1. Results of Meat Quality Assessment

No statistically significant differences were found in the analysed physical parameters of meat, nor in the contents of water, fat, and collagen between the groups (*p* > 0.05) (Table 2).

The meat of boars had a high protein content (average 23%). A higher protein level was determined in the meat of boars from group E1 compared to those from group E2 (*p* ≤ 0.01), as well as in the meat of boars from group E1 compared to those from group C (*p* ≤ 0.01).

Table 3 presents the fatty acid profile in the meat of the boars.

Analysis of the fatty acid content in boar meat showed no significant differences between the groups except for n-6 PUFA, which was significantly higher in group C than in group E2 (*p* ≤ 0.05). The AI value ranged from 0.56 (groups C and E1) to 0.59 (group E2), whereas the thrombogenic index value was the highest in the meat of boars from group E2.

### 3.2. Results of Backfat Quality Assessment

Table 4 presents the results of selected physicochemical characteristics of backfat. No statistically significant differences were found between the groups in terms of all tested parameters of backfat colour and water content (*p* > 0.05).

The highest total protein content was determined in the backfat of boars from group E1, and it was higher by 1.50 percentage points compared to the control boars (*p* ≤ 0.05). Statistically significant differences were also observed in backfat content between the groups. The highest fat level was recorded in group C, which was 4.19 percentage points higher compared to group E1 and 5.34 percentage points higher compared to group E2 (*p* ≤ 0.05).

The analysis of the fatty acid profile in backfat also revealed statistically significant differences between the studied groups (Table 5).

A higher content of saturated fatty acids (SFAs) was determined in the backfat of boars from groups C and E1 compared to those from group E2 (*p* ≤ 0.01). The lowest level of C18:0 was found in the backfat of boars from group E2 compared to the animals from groups E1 and C (*p* ≤ 0.01). The level of C16:0 in the backfat of boars from group E2 differed statistically significantly compared to those from groups E1 and C (*p* ≤ 0.05). Significant differences between the groups were confirmed in the content of individual polyunsaturated fatty acids (PUFAs): C18:3 n-3, C20:4 n-6, C22:2, and C22:6. The highest content of C18:3 n-3 was found in group E2. The differences compared to the other groups were statistically significant at *p* ≤ 0.05 (group E1) and *p* ≤ 0.01 (group C). The level of C20:4 n-6 acid differed significantly between groups C and E1 (*p* ≤ 0.05), whereas the content of C22:2 acid was the lowest in backfat of boars from group E2, being significantly lower than in group E1 (*p* ≤ 0.01) and group C (*p* ≤ 0.05). A higher content of C22:6 acid was determined in the backfat of pigs from group C compared to those from group E2 (*p* ≤ 0.05). The content of n-3 polyunsaturated fatty acids (n-3 PUFAs) was significantly higher in group E2 than in group C (*p* ≤ 0.01). The PUFA n-6/n-3 ratio was significantly lower in the uncastrated boars with a slaughter weight of 105 kg compared to the immunologically castrated boars (*p* ≤ 0.05). The TI value was significantly lower in group E2 compared to groups C and E1 (*p* ≤ 0.05).

### 3.3. Compounds Responsible for Boar Taint

Due to high variability and undetectable contents of the tested compounds, we examined indole, skatole, androstenol, and androsterone in meat and backfat from boars, and Table 6 and Table 7 show their minimum, maximum, average, and median values. Statistical analysis was performed only for compounds detected in at least six samples per group.

The highest average indole content was determined in the meat of boars from group C, whereas the lowest one was in the meat of boars from group E1. A similar trend was observed for skatole, with the highest average content found in the meat of boars from group C and the lowest in those from group E1, where the level of this compound was undetectable in one sample. An opposite observation was made for the androstenol content, with the lowest mean value determined in group C and the highest in group E1 (*p* ≤ 0.05). Group E2 showed the highest average androsterone content, with undetectable levels in three samples. The average androsterone level in the meat of boards from group C was the lowest, with undetectable levels in six samples. In the meat of boars from group E1, androsterone was undetectable in two samples.

Six samples from group C of boars had undetectable indole levels. The average content of this compound in the remaining samples was lower than in groups E1 and E2. The indole level was also undetectable in six samples from group E2, and the average of the remaining samples was the highest compared to groups C and E1. In group E1, there were five samples with undetectable levels of indole. In the case of skatole, its level was undetectable in all samples from both group C and group E1. In group E2, it was detectable in only one sample. Androstenol was detected in every sample of backfat from all groups. Its highest average content was found in group E1, and the lowest in the control group (*p* ≤ 0.05). In turn, the content of androsterone was determined in only one sample from the control group, while in the remaining samples, its level was undetectable. The highest average androsterone content was determined in the backfat of boars from group E1, with an undetectable content in one sample. The lowest average was found in group E2, with three undetectable samples.

Based on the results obtained using the Heracles II electronic nose, various volatile compounds was identified in both meat and backfat, with differences observed between the experimental groups (Table 8 and Table 9).

In the case of meat (Table 8), the highest number of volatile compounds was detected in group E1 (24 compounds), followed by group E2 (19), and the control group C (15). These compounds belonged to various chemical classes, such as esters, alcohols, aldehydes, furans, thiols, terpenes, and amines. Their sensory profiles included both desirable aromas (e.g., fruity, green) and potentially undesirable ones (e.g., animal, burnt, sour). The presence of a greater number of volatile compounds in the meat of non-castrated boars may indicate a more diverse aroma profile of the raw material in this experimental group.

Differences were also observed in the volatile compound profile of backfat (Table 9). The highest number of identified compounds was found in group E2 (19), compared to 16 in group C and 15 in group E1, and they included alcohols (e.g., 1-hexanol, 2-octanol), aldehydes, amines, and carboxylic acids. Some of these compounds were associated with undesirable sensory attributes, such as onion, fishy, or rancid odours.

Table 10 presents changes in relative peak surface areas corresponding to the presence of indole in meat and backfat. Statistical analysis revealed significant differences between the groups in both meat and backfat. In meat, the greatest indole peak areas were observed in the control group, with significantly lower values recorded in groups E1 and E2. An opposite trend was noted in backfat, where the greatest peak surface area was determined in group E2, being significantly higher than in group E1 and group C.

**Figure 1:**
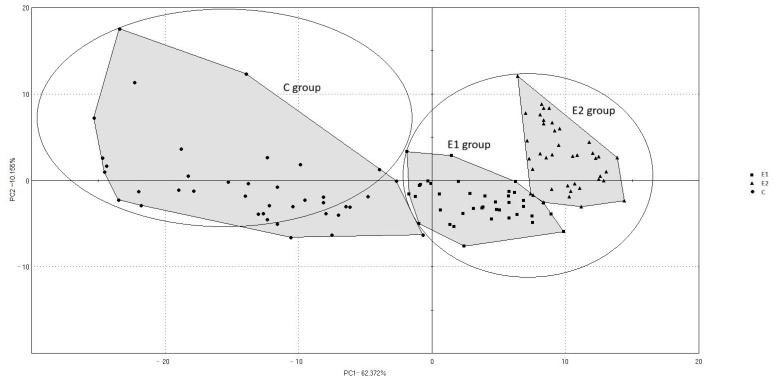
Principal component analysis (PCA) based on volatile compounds detected in meat samples from groups C, E1, and E2.

The map in Figure 1 presents the distribution of individual meat samples across the first two principal components (PC1: 62.37%, PC2: 10.15%), which together explained 72.53% of the total variance. Samples were grouped according to the experimental group (C, E1, E2), with ellipses indicating the spatial dispersion of each group. The separation between groups suggests significant differences in the volatile compound profiles, particularly between group C and the experimental groups.

The principal component analysis (PCA) presented on Figure 1 distinctly separated the three experimental groups of pigs (C, E1, and E2) along the first principal component (PC1: 62.37% of the total variance), demonstrating clear differentiation in the profiles of volatile compounds. Immunologically castrated boars from the control group (C) were characterised by the exclusive presence of terpenes, such as α-pinene and 1,8-cineole, responsible for hay-like and herbaceous odour. The E1 group exhibited a unique presence of furans (2-methylfuran, 5-methylfurfural) and pyrrole (2-propionylpyrrole), which contributed to burnt, acidic, and roasted sensory impressions. In addition, esters (methyl isobutyrate) imparted fruity notes, while phenolic compounds (2,6-dimethoxyphenol) provided smoky and phenolic nuances. The detection of heptyl mercaptan and propylene glycol further introduced sulphurous and alcoholic components to the overall aroma profile. In contrast, the E2 group was characterised by the presence of 2,3-butanediol, octane, p-menthatriene, and (E,E)-2,4-nonadienal, associated with bitter, woody, and cereal odour notes. These compounds contributed to a more balanced and mild overall aroma profile compared with the other groups. Overall, the PCA separated immunocastrated and uncastrated boars primarily along PC1, reflecting a clear transition from terpene-dominated profiles, typical of uncastrated boars, toward aldehyde and ester profiles characteristic of immunocastrated pigs.

**Figure 2:**
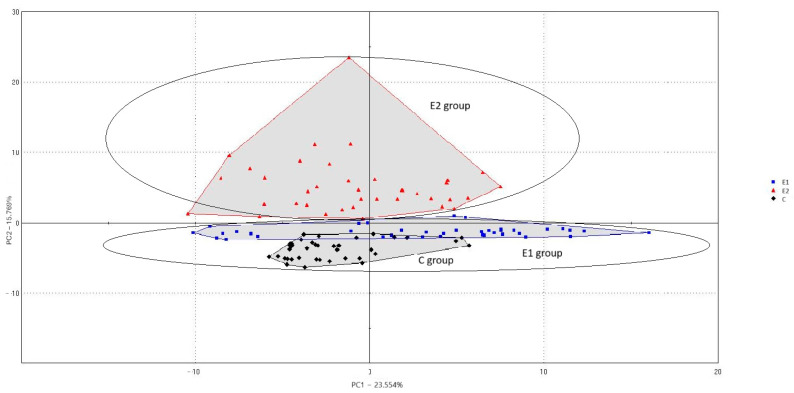
Principal component analysis (PCA) based on volatile compounds detected in backfat samples from groups C, E1, and E2.

The PCA biplot (Figure 2) shows the distribution of backfat samples in a two-dimensional space defined by PC1 (23.55%) and PC2 (15.76%), jointly explaining 39.31% of the total variance. Distinct groupings of the samples are visible, with partial overlap between groups C and E1, and clearer separation from group E2. The spatial configuration of the groups reflects the variability in the volatile compound profile of the backfat. Fat obtained from boars from the control group (C) was characterised by the presence of alcohols (1-propanol, 2-octanol) and terpene (terpinolene), as well as aldehyde (but-2-enal). These compounds contributed to alcoholic, fatty, green, and anisic odour notes, which are commonly associated with the lipid oxidation processes. The E1 group exhibited unique volatile components, including formic acid and hexanoic acid (carboxylic acids with pungent and fatty notes), 1-butanamine (amine with a fishy odour), and sotolon (a lactone associated with mushroom aroma). This combination indicates the presence of compounds contributing to more intense, acidic, and umami-like sensory characteristics. In contrast, the E2 group was characterised by 2-furanmethanol, 1-hexanol, heptyl mercaptan, undecane, and p-menthatriene, providing bread-like, fatty, onion, woody, and alkane notes.

## 4. Discussion

### 4.1. Quality of Meat

Analysis of physicochemical parameters in the present study showed that there were no statistically significant differences between the groups in any physical parameters of meat (colour, drip loss, cooking loss, WHC). Pauly et al. (2009) [26] and Škrlep et al. (2020) [27] also observed no differences in meat colour (on the CIE scale) between immunocastrated and uncastrated boars. In contrast, Gispert et al. (2010) [28] and dos Santos et al. (2021) [29] showed a lower L* value for the meat of uncastrated males compared to immunocastrates, but did not report significant differences in a* and b* values. Aluwé et al. (2013) [30] observed that the colour of boar meat had a higher b* value compared to immunocastrates, with no differences in L* and a* values. Corino et al. (2009) [31] and Ba et al. (2019) [32] studied the effect of slaughter weight on the physicochemical parameters of meat. They demonstrated increased red and yellow hue in the colour assessment of meat obtained from pigs with a higher body weight at slaughter. Greater losses in cooking loss for the meat of immunologically castrated boars compared to the meat of boars were observed by Aluwé et al. (2013) [30] and dos Santos et al. (2021) [29]. This finding is, however, inconsistent with observations made by Škrlep et al. (2020) [27], who found no statistically significant differences between boar meat and immunocastrated boar meat in terms of both cooking loss and drip loss values. Ba et al. (2019) [32] observed increased cooking losses in meat from pigs slaughtered at a lower body weight (100 kg) compared to the pigs with a slaughter weight of 120 kg, whereas Corino et al. (2009) [31] did not observe such differences in meat of pigs slaughtered at 120 kg and 160 kg. WHC is a particularly important indicator of meat quality for technological and economic reasons. Its level is influenced by many factors, such as protein content, pH, pre-slaughter handling, species and sex of the animal, slaughter technique, and the technological process itself [33]. Although no statistically significant differences were observed in the present study, a tendency for reduced WHC was noted in the meat of lighter boars slaughtered at a body weight of 105 kg. Different results were reported by Ba et al. (2019) [32], who observed a decreased water-holding capacity in meat with an increasing slaughter weight of pigs.

The present study showed statistically significant differences in the total protein content of meat between the groups studied, with no differences in water, fat, and collagen levels. Collagen is an important component that determines meat tenderness and, thus, influences consumers’ perception of its palatability. It also provides muscles with adequate mechanical strength [34]. From a nutritional point of view, collagen protein is considered a complete protein due to the lack of tryptophan [35]. Škrlep et al. (2019) [36] showed a significantly higher collagen content in boar meat compared to the meat of castrated boars. In the present study, meat obtained from boars slaughtered at a body weight of 120 kg was characterised by a higher protein content, which may be related to both slaughter weight and hormonal status. This is, however, inconsistent with findings from the study by Ba et al. (2019) [32], who showed that the meat of pigs with a slaughter weight of 120 kg had a lower protein content and a higher fat content compared to the meat of pigs slaughtered at 100 kg. In the experiments of Latorre et al. (2004) [37] and Corino et al. (2009) [31], slaughter weight did not have a significant effect on meat protein content. The effect of immunocastration on the chemical composition of meat was studied by Grela et al. (2020) [38] and Božičković et al. (2025) [39]. Grela et al. (2020) [38] observed no significant differences in the chemical composition of meat from immunocastrates vs. uncastrated native breed boars, and Božičković et al. (2025) [39] also did not confirm the effect of early and late immunocastration on the chemical composition of meat compared to uncastrated boars.

### 4.2. Quality of Backfat

The analysis of backfat colour in the present study did not reveal any statistically significant differences between the groups. However, statistically significant differences were found in the protein and fat contents of the backfat. The protein content in backfat, regardless of the group, was relatively high compared to the results reported by other authors [40,41,42]. The highest level was observed in the backfat of heavier uncastrated boars. This may indicate a higher content of connective tissue, which is consistent with reports of a lower fat content in uncastrated males [43,44]. A high protein content in backfat is undesirable, as it may reduce the use value of the raw material [40,41]. Backfat obtained from pigs of all groups had a high water content, averaging 27.87%. The water content in the backfat of uncastrated males was slightly higher than in the meat of the immunologically castrated boars. A higher water content in backfat may increase its susceptibility to the development of pathogenic microflora and increase its tendency to become rancid [40].

### 4.3. Fatty Acid Profile in Meat and Backfat

Fat and fatty acids, both in the adipose tissue and muscles, have a significant impact on various aspects of meat quality and are crucial for its nutritional value [45].

The nutritional value and sensory qualities of pork are determined by the interactions between saturated fatty acids (SFAs), monounsaturated fatty acids (MUFAs), and polyunsaturated fatty acids (PUFAs). Increasing consumer expectations regarding the taste, tenderness and juiciness of meat, coupled with concerns over nutritional recommendations, urge the need to seek an optimal balance between these requirements [46].

The proportion of energy available for fat deposition in pigs increases during fattening, which translates into an increase in the rate of de novo fatty acid synthesis [47]. The present study showed that meat and backfat obtained from boars slaughtered at a body weight of 120 kg had a higher proportion of SFA than the meat of boars slaughtered at 105 kg. Similar findings were reported in the research by Skiba et al. (2013) [48], where the contents of the major acids of this group, i.e., C16:0 and C18:0 acids, were lower in meat and backfat from animals slaughtered at a body weight of 105 kg compared to 120 kg. In the study by Skiba et al. (2013) [48], the C16:0 acid content increased with the increase in the body weight of the animals, while the C18:0 acid level remained unchanged.

A lower slaughter weight of animals translates into their thinner backfat, and thus a lower content of monounsaturated fatty acids (MUFAs) stored in the adipose tissue as a result of de novo synthesis [49]. The present study results confirm this thesis. The backfat and meat of the animals from group E2 had a lower content of MUFA in than those of the animals with a slaughter weight of 120 kg. Similar results were also reported by Pauly et al. (2009) [26].

Omega-3 and omega-6 fatty acids play an important role in human health due to their therapeutic potential in the treatment of chronic diseases [50]. Proper functioning of the body requires a balance between omega-6 and omega-3 acids. Pork usually has a less favourable ratio of omega-6 to omega-3 acids than the ideal ratio (1:1 to 5:1) [51]. In the present study, this ratio in pork was almost 10:1, while in backfat, it was closer to the recommended 5:1. In turn, Grela et al. (2013) [52] showed an even higher ratio of omega-6 to omega-3 fatty acids in meat and backfat compared the present study.

AI and TI indices play a key role in determining the nutritional value of pork. In this study, their values were favourably low (in meat and backfat), regardless of slaughter weight and castration status. A lower AI value translates into reduced lipid binding to the endothelium and the formation of atherosclerotic plaques in blood vessels. On the other hand, a lower TI index reduces the risk of blood clotting disorders and thrombus formation. The AI of pork is subject to fluctuations due to factors such as the origin of a particular cut, the processing techniques used, and the diet of farm animals [53].

### 4.4. Contents of Indole, Skatole, Androstenol, and Androsterone in Meat and Backfat

Analysis of compounds responsible for boar taint showed reduced levels of androstenol and androsterone in the meat of boars from the control group. Furthermore, the level of androsterone in meat was undetectable in 6 out of the 10 samples. Similar observations were made for backfat, with the average content of androstenol also being the lowest in the immunocastrated group, and androsterone undetectable in as many as nine samples. The reduced hormone levels in meat and backfat obtained from the control boars confirm that immunocastration was performed correctly. The effectiveness of Improvac^®^ in reducing the sex steroids responsible for boar taint has been confirmed by many authors [54,55,56,57]. However, it is essential to note that high efficacy does not necessarily imply reliability, and factors that may limit its effectiveness should be taken into account. The literature reports isolated cases of individuals showing unsatisfactory results of immunocastration [58,59,60,61]. It is, however, difficult to determine unambiguously whether a true lack of response to immunocastration occurred or whether the preparation was administered incorrectly [61]. Werner et al. (2021) [62] observed that early immunocastration, carried out already at the piglet production stage, did not eliminate boar taint in all males. Another factor that may influence the occurrence of boar taint in castrated individuals is intestinal infection [63]. In the study by Weiler et al. (2013) [64], immunocastration was shown to increase the feed intake rate. In immunocastrated males exhibiting this phenomenon, higher indole concentrations were recorded, which the authors explained by changes in gastrointestinal pH that promote the development of indole-producing microorganisms. In the present study, the lowest mean indole content was determined in the backfat of boars from group C, whereas skatole was not detected in any of the samples tested in this group. The effect of immunocastration in reducing skatole and indole levels in backfat was confirmed by Zamaratskaia et al. (2008) [65]. However, Needham et al. (2020) [66] did not observe any reduction in indole content in the adipose tissue linked to the immunocastration, but they did observe a reduction in skatole. Lower levels of skatole in the adipose tissue of immunologically castrated boars were also reported by Škrlep et al. (2010) [67], Aleksić et al. 2012 [68], and Stupka et al. (2017) [69]. Han et al. (2019) [70] suggested that, by reducing the formation of skatole in the intestines and simultaneously accelerating its degradation metabolism in the liver, immunocastration impairs skatole accumulation in the adipose tissue, and that the reduced formation of skatole in the intestines may result from the suppressed effect of insulin-like growth factor 1 (IGF1) on the renewal of the mucous membrane of the ileum and colon. Chen et al. (2006) [71] used human chorionic gonadotropin (hCG) in their experiment and found that sex steroids also affected indole metabolism. In contrast, the present study revealed different trends from meat compared to backfat. While immunocastration effectively reduced sex steroid concentrations and decreased indole levels in backfat, the highest average concentrations of indole and skatole were observed in meat obtained from immunocastrated boars. This suggests that the mechanisms controlling indole and skatole deposition in lean tissue may differ from those in adipose tissue. Meinert et al. (2017) [72] found correlations between skatole levels in adipose tissue and meat; however, these findings were not supported by our data. The contents of indole compounds in the lean tissue may be affected by the thickness of fat covering the muscle being tested [73]. Repeated analyses of backfat with undetectable levels of skatole in the present study may indicate that the animals were kept in good hygienic conditions [74]. According to Kjeldsen (1993) [75], a wet feed mixture and unlimited access to water also reduce the skatole content of fat, which is consistent with the study by Czech et al. (2022) [76], in which castrated boars and immunologically castrated boars administered a wet feed mixture had a lower skatole content in meat and fat than those fed a dry feed mixture. A diet high in fibre and carbohydrates that are not broken down in the intestines reduces skatole production [77]. The indole contents determined in meat and backfat by gas chromatography were consistent with the results of the compound profile determined using an electronic nose. In the analyses performed using both methods, the highest content of indole was determined in the meat of boars from group C, and the lowest in the meat of boars from group E1. In backfat, however, the highest level was found in group E2 and the lowest in group C. The results obtained indicate that despite the lack of distinct quantitative differences in indole content, its contribution to the odour perception of meat and fat differed significantly between groups, as detected by e-nose analysis.

## 5. Conclusions

In summary, this study compared the quality of meat and fat from immunologically castrated boars and uncastrated boars with slaughter weights of 120 and 105 kg. The chemical compositions of the meat and quality parameters were comparable in all groups, with only a higher protein content in the meat of heavier uncastrated boars and a higher content of n-6 PUFA acids in the meat from immunologically castrated males. The omission of immunocastration negatively affected backfat quality by increasing its protein content and decreasing its fat content. Both the slaughter weight and immunocastration status influenced the fatty acid profile in backfat. The analysis of compounds responsible for boar taint confirmed the effectiveness of immunocastration in reducing hormone levels (androstenol, androsterone) in meat and backfat. However, variability in indole concentration was observed. Analysis using an electronic nose showed the highest indole content in meat and the lowest in fat from immunologically castrated males, indicating that the effect of immunocastration was not uniform in all tissues. The significant differences in the levels of the compounds tested (indole, skatole, androstenol, and androsterone) may reflect the influence of individual variability. The results obtained suggest that despite the effective reduction in sex steroids as a result of immunocastration, in some cases, individually varying levels of compounds responsible for boar taint, especially indole, may persist.

## Figures and Tables

**Table 1:** Experimental design.

Item	Groups		
	C	E1	E2
Number of animals, heads	10	10	10
Castration	immunological	none	none
Slaughter weight, kg	120.0	120.0	105.0

**Table 2:** Selected physicochemical parameters of meat.

Item	Groups			*p*-Value
	C	E1	E2	
Colour of meat				
L*	49.96 ± 2.29	48.86 ± 1.33	49.92 ± 1.96	0.353
a*	5.22 ± 1.02	5.18 ± 0.89	4.96 ± 1.11	0.818
b*	4.58 ± 1.76	4.71 ± 0.81	4.28 ± 1.05	0.342
Drip loss, %	2.86 ± 0.85	2.53 ± 0.95	2.47 ± 0.66	0.698
Cooking loss, %	17.90 ± 1.90	17.34 ± 2.12	18.37 ± 1.40	0.459
WHC *, cm^2^/g	21.63 ± 3.38	21.63 ± 3.30	24.54 ± 2.84	0.080
Chemical composition of meat				
Water	72.86 ± 0.77	72.39 ± 0.59	73.11 ± 0.73	0.101
Protein	22.87 ^A^ ± 0.17	23.48 ^B^ ± 0.45	22.99 ^A^ ± 0.29	0.001
Fat	3.70 ± 0.57	3.83 ± 0.58	3.43 ± 0.65	0.505
Collagen	1.13 ± 0.10	1.04 ± 0.13	1.22 ± 0.23	0.059

WHC *—water holding capacity; A, B—mean values marked with different letters differ significantly (*p* ≤ 0.01).

**Table 3:** Fatty acid profile in meat.

Item	Groups			*p*-Value
	C	E1	E2	
C14:0	1.32 ± 0.01	1.32 ± 0.03	1.29 ± 0.10	0.683
C16:0	24.08 ± 0.21	23.97 ± 0.30	23.76 ± 2.39	0.602
C16:1	2.43 ± 0.04	2.43 ± 0.07	2.34 ± 0.16	0.355
C18:0	14.77 ± 0.20	14.79 ± 0.24	14.59 ± 2.08	0.600
C18:1	36.98 ± 0.31	36.82 ± 0.62	35.04 ± 3.76	0.183
C18:2 n-6	4.67 ± 0.19	4.59 ± 0.23	4.31 ± 0.43	0.051
C18:3 n-3	0.44 ± 0.02	0.45 ± 0.01	0.43 ± 0.06	0.654
C20:1	0.03 ± 0.01	0.03 ± 0.01	0.03 ± 0.01	0.304
C20:4 n-6	0.53 ± 0.03	0.54 ± 0.05	0.50 ± 0.08	0.552
C22:1	0.19 ± 0.01	0.20 ± 0.02	0.20 ± 0.03	0.864
C22:2	0.17 ± 0.11	0.11 ± 0.31	0.16 ± 0.09	0.317
C22:5	0.03 ± 0.01	0.03 ± 0.25	0.03 ± 0.02	0.395
C22:6	0.03 ± 0.02	0.04 ± 0.02	0.03 ± 0.01	0.068
SFA	40.16 ± 0.37	40.07 ± 0.43	39.63 ± 4.52	0.870
MUFA	39.63 ± 0.34	39.42 ± 0.66	37.61 ± 3.90	0.169
PUFA	5.43 ± 0.24	5.32 ± 0.22	5.02 ± 0.50	0.109
PUFA n-3	0.50 ± 0.03	0.53 ± 0.03	0.48 ± 0.06	0.339
PUFA n-6	5.21 ^a^ ± 0.19	5.13 ^ab^ ± 0.19	4.81 ^b^ ± 0.49	0.039
PUFA n-6/n-3	10.40 ± 0.97	9.82 ± 0.75	9.99 ± 0.66	0.374
AI	0.56 ± 0.01	0.56 ± 0.01	0.59 ± 0.10	0.567
TI	1.67 ± 0.01	1.67 ± 0.02	1.76 ± 0.31	0.340

a, b—mean values marked with different letters differ significantly (*p* ≤ 0.05).

**Table 4:** Assessment of selected physicochemical traits of backfat.

Item	Groups			*p*-Value
	C	E1	E2	
Colour of backfat				
L*	77.69 ± 1.40	77.85 ± 1.48	76.60 ± 1.60	0.147
a*	3.41 ± 1.40	3.52 ± 1.38	3.77 ± 0.66	0.789
b*	5.84 ± 1.54	6.21 ± 0.95	6.49 ± 0.95	0.480
Chemical composition of backfat				
Water	25.02 ± 3.68	27.70 ± 3.38	30.89 ± 6.29	0.072
Protein	4.70 ^a^ ± 0.43	6.20 ^b^ ± 1.55	5.69 ^ab^ ± 1.23	0.025
Fat	70.09 ^a^ ± 3.36	65.90 ^b^ ± 5.57	64.75 ^b^ ± 4.32	0.033

a, b—mean values marked with different letters differ significantly (*p* ≤ 0.05).

**Table 5:** Fatty acid profile in backfat.

Item	Groups			*p*-Value
	C	E1	E2	
C14:0	1.21 ± 0.08	1.21 ± 0.05	1.20 ± 0.04	0.792
C16:0	22.67 ^a^ ± 0.87	22.62 ^a^ ± 0.70	21.67 ^b^ ± 0.70	0.027
C16:1	2.13 ± 0.21	2.02 ± 0.22	2.17 ± 0.21	0.310
C18:0	13.43 ^A^ ± 0.57	13.49 ^A^ ± 0.89	12.21 ^B^ ± 0.75	0.003
C18:1	35.76 ± 1.24	35.47 ± 1.41	34.47 ± 0.87	0.064
C18:2 n-6	3.96 ± 0.33	4.21 ± 0.16	4.29 ± 0.26	0.059
C18:3 n-3	0.67 ^A^ ± 0.06	0.70 ^a^ ± 0.08	0.83 ^Bb^ ± 0.07	0.002
C20:1	0.03 ± 0.01	0.02 ± 0.01	0.03 ± 0.01	0.714
C20:4 n-6	0.48 ^a^ ± 0.19	0.24 ^b^ ± 0.05	0.36 ^ab^ ± 0.15	0.016
C22:1	0.18 ^ab^ ± 0.03	0.16 ^a^ ± 0.03	0.19 ^b^ ± 0.02	0.019
C22:2	0.28 ^a^ ± 0.10	0.36 ^A^ ± 0.19	0.16 ^Bb^ ± 0.12	0.009
C22:5	0.03 ± 0.02	0.05 ± 0.02	0.05 ± 0.02	0.225
C22:6	0.06 ^a^ ± 0.02	0.05 ^ab^ ± 0.02	0.03 ^b^ ± 0.02	0.033
SFA	37.32 ^A^ ± 1.15	37.32 ^A^ ± 1.57	35.08 ^B^ ± 1.34	0.003
MUFA	38.10 ± 1.25	37.67 ± 1.52	36.85 ± 0.97	0.083
PUFA	4.81 ± 0.26	4.91 ± 0.29	4.89 ± 0.40	0.727
PUFA n-3	0.76 ^A^ ± 0.08	0.80 ^AB^ ± 0.09	0.91 ^B^ ± 0.07	0.003
PUFA n-6	4.44 ± 0.17	4.45 ± 0.15	4.65 ± 0.32	0.088
PUFA n-6/n-3	5.90 ^a^ ± 0.62	5.62 ^ab^ ± 0.57	5.17 ^b^ ± 0.72	0.020
AI	0.55 ± 0.03	0.56 ± 0.03	0.54 ± 0.02	0.447
TI	1.57 ^a^ ± 0.07	1.58 ^a^ ± 0.10	1.48 ^b^ ± 0.08	0.031

a, b—mean values marked with different letters differ significantly (*p* ≤ 0.05); A, B—mean values marked with different letters differ significantly (*p* ≤ 0.01).

**Table 6:** Indole, skatole, androstenol, and androsterone content in meat (ng/g).

Item		Groups			*p*-Value
		C	E1	E2	
Indole	Undetectable Average Minimum Maximum Median	- 43.7 ± 20.61 18.4 80.2 39.9	- 32.7 ± 20.21 10.9 76.8 28.8	- 33.0 ± 12.06 10.9 48.9 32.8	0.301
Skatole	Undetectable Average Minimum Maximum Median	- 19.1 ± 13.12 3.7 40.7 16.6	1 9.9 ± 4.36 4.3 17.4 10.4	- 11.5 ± 6.20 1.2 19.8 10.9	0.289
Androstenol	Undetectable Average Minimum Maximum Median	- 59.6 ^a^ ± 9.50 49.7 78.0 56.4	- 70.9 ^b^ ± 13.29 57.4 96.2 66.8	- 67.0 ^ab^ ± 10.41 55.7 88.2 64.2	0.025
Androsterone	Undetectable Average Minimum Maximum Median	6 120.7 25.1 169.7 143.9	2 141.3 40.8 331.7 136.5	3 147.6 43.6 386.7 112.4	-

a, b—mean values marked with different letters differ significantly (*p* ≤ 0.05).

**Table 7:** Indole, skatole, androstenol, and androsterone content in backfat (ng/g).

Item		Groups			*p*-Value
		C	E1	E2	
Indole	Undetectable Average Minimum Maximum Median	6 11.0 9.6 12.4 11.0	5 13.0 10.0 14.6 13.5	6 53.3 11.9 171.6 14.8	-
Skatole	Undetectable Average Minimum Maximum Median	10 -	10 -	9 405.0	-
Androstenol	Undetectable Average Minimum Maximum Median	- 493.0 ^a^ ± 193.77 307.9 974.7 457.9	- 996.6 ^b^ ± 623.01 309.4 2214.1 816.5	- 779.2 ^ab^ ± 517.30 362.8 2007.8 573.9	0.018
Androsterone	Undetectable Average Minimum Maximum Median	9 6823.1	1 6017.8 1463.2 13,475.1 3321.0	3 4646.7 203.9 12,294.1 3278.1	-

a, b—mean values marked with different letters differ significantly (*p* ≤ 0.05).

**Table 8:** Volatile compound profile in meat.

Possibly Matching Compounds	IR KMXT-5 *	Chemical Groups of Compounds	Sensory Descriptors	Groups		
				C	E1	E2
methyl formate	372	ester	agreeable	X	X	X
ethanol	450	alcohol	alcoholic	X	X	X
propanal	491	aldehyde	earthy	X	X	X
1-propanol	521	alcohol	alcoholic	X	X	X
2-mercaptoethanol	558	thiol	sulphurous		X	X
2-methylfuran	584	furan	burnt		X	
but-(2)-enal	635	aldehyde	green	X	X	X
3-methylbutanal	652	aldehyde	fatty		X	X
methyl isobutyrate	665	ester	fruity		X	
2-ethyl furan	704	furan	acidic	X	X	X
methyl butanorate	734	ester	ester	X	X	
propylenglycol	754	alcohol	alcoholic		X	
2-methylpentanal	762	aldehyde	earthy	X	X	X
2,3-butanediol	790	diol	bitter			X
octane	825	alkane	alkane			X
e-2-hexen-1-ol	848	alcohol	green		X	
1-hexanol	876	alcohol	fatty		X	X
pentanoic acid	905	acid	rancid	X	X	X
5-methylfurfural	956	furan	acidic		X	
2-(2-ethoxyethoxy)ethanol	1001	ether	mild		X	X
pinene	1003	terpen	hay	X		
heptyl mercaptan	1026	thiol	sulphurous		X	
benzeneacetaldehyde	1028	aldehyde	grassy	X		X
1,8-cineole	1046	terpene	herbaceous	X		
2-propionylpyrrole	1049	pyrrole	roast		X	
undecane	1076	alkane	alkane		X	
gamma-terpinene	1078	terpen	etheral	X		X
p-menthatriene	1131	terpene	woody			X
2,6-dimethoxy-phenol	1203	phenol	phenolic		X	
(e, e)-2,4-nonadienal	1208	aldehyde	cereal			X
decanal	1229	aldehyde	fatty	X	X	
indole	1295	amine	animal	X	X	X
Total				15	24	19

* IR MXT-5—retention indexes for MXT-5 column; X—presence of the given volatile compound in the profile of each group.

**Table 9:** Volatile compound profile in backfat.

Possibly Matching Compounds	IR KMXT-5 *	Chemical Groups of Compounds	Sensory Descriptors	Groups		
				C	E1	E2
methyl formate	373	ester	agreeable	X	X	X
trimethylamine	420	amine	ammoniacal		X	X
ethanol	451	alcohol	alcoholic,	X	X	X
propanal	492	aldehyde	acetaldehyde	X	X	X
2-methylpropanal	521	aldehyde	aldehydic	X	X	X
1-propanol	552	alcohol	alcoholic	X		X
formic acid	565	carboxylic acid	pungent		X	
1-propanol, 2-methyl-	628	alcohol	bitter	X	X	X
1-butanamine	635	amine	fishy		X	
but-(2)-enal	652	aldehyde	green	X		
pent-1-en-3-ol	677	alken, alkohol	meaty	X		X
2-ethyl furan	704	furan	pungent	X	X	X
2-methylpentanal	763	aldehyde	earthy	X	X	X
2-furanmethanol	869	alcohol	bread			X
1-hexanol	877	alcohol	fatty			X
nonane	904	alkane	alkane	X	X	X
2-octanol	1000	alcohol	fatty	X		
hexanoic acid	1004	carboxylic acid	fatty		X	
heptyl mercaptan	1028	thiol	onion			X
1,8-cineole	1045	ether	herbaceous	X	X	X
terpinolene	1077	terpene	anisic	X		
undecane	1081	alkane	alkane			X
p-menthatriene	1130	terpene	woody			X
sotolon	1134	lactone	mushroom		X	
pyridine, 2-pentyl-	1205	heteroaromatic compound	tallow	X		X
indole	1296	amine	animal	X	X	X
Total				16	15	19

* IR MXT-5—retention indexes for MXT-5 column; X—presence of the given volatile compound in the profile of each group.

**Table 10:** Changes in relative surface areas of peaks of indole in meat and backfat.

Indole	Groups			*p*-Value
	C	E1	E2	
Meat	7.63 ^A^ ± 2.46	3.86 ^B^ ± 1.15	4.29 ^B^ ± 1.16	0.001
Backfat	3.74 ^A^ ± 1.07	4.05 ^A^ ± 0.99	5.28 ^B^ ± 1.29	0.001

A, B—mean values marked with different letters differ significantly (*p* ≤ 0.01).

## Data Availability

The data that support the findings of this study are available on request from the corresponding author (A.Z.).

## References

[1] Heyrman E., Millet S., Tuyttens F.A.M., Ampe B., Janssens S., Buys N., Wauters J., Vanhaecke L., Aluwé M. (2021). On-Farm Prevalence of and Potential Risk Factors for Boar Taint. Animal.

[2] Duarte D.A.S., Schroyen M., Mota R.R., Vanderick S., Gengler N. (2021). Recent Genetic Advances on Boar Taint Reduction as an Alternative to Castration: A Review. J. Appl. Genet..

[3] Squires E.J., Bone C., Cameron J. (2020). Pork Production with Entire Males: Directions for Control of Boar Taint. Animals.

[4] Hess R.A., Park C.J., Soto S., Reinacher L., Oh J.E., Bunnell M., Ko C.M.J. (2024). Male Animal Sterilization: History, Current Practices, and Potential Methods for Replacing Castration. Front. Vet. Sci..

[5] Aluwé M., Heyrman E., Almeida J.M., Babol J., Battacone G., Čítek J., Furnols M.F.I., Getya A., Karolyi D., Kostyra E. (2020). Exploratory Survey on European Consumer and Stakeholder Attitudes towards Alternatives for Surgical Castration of Piglets. Animals.

[6] Hay M., Vulin A., Génin S., Sales P., Prunier A. (2003). Assessment of Pain Induced by Castration in Piglets: Behavioral and Physiological Responses over the Subsequent 5 Days. Appl. Anim. Behav. Sci..

[7] Breitenlechner A., Bünger M., Ruczizka U.K., Dolezal M., Auer U., Buzanich-Ladinig A. (2024). Comparison between Intramuscular and Intranasal Administration of Sedative Drugs Used for Piglet Castration. Animals.

[8] Hokkanen A.H., Coutant M., Heinonen M., Norring M., Adam M., Oliviero C., Bergqvist T., Valros A. (2025). Two Restraining Devices in Connection to Surgical Castration with or without Local Anesthesia: Effects on Piglet Stress. Porc. Health Manag..

[9] Pesenti Rossi G., Dalla Costa E., Filipe J.F.S., Mazzola S.M., Motta A., Borciani M., Gastaldo A., Canali E., Pilia F., Argenton M. (2022). Does Immunocastration Affect Behaviour and Body Lesions in Heavy Pigs?. Vet. Sci..

[10] Pejsak Z., Truszczyński M. (2009). Immunologiczna Kastracja Knurków. Życie Weter..

[11] Čandek-Potokar M., Škrlep M., Zamaratskaia G., Čandek-Potokar M., Škrlep M., Zamaratskaia G. (2017). Immunocastration as Alternative to Surgical Castration in Pigs. Theriogenology.

[12] Kress K., Millet S., Labussière É., Weiler U., Stefanski V. (2019). Sustainability of Pork Production with Immunocastration in Europe. Sustainability.

[13] von Borell E., Bonneau M., Holinger M., Prunier A., Stefanski V., Zöls S., Weiler U. (2020). Welfare Aspects of Raising Entire Male Pigs and Immunocastrates. Animals.

[14] Škrlep M., Tomašević I., Mörlein D., Novaković S., Egea M., Garrido M.D., Linares M.B., Peñaranda I., Aluwé M., Font-I-furnols M. (2020). The Use of Pork from Entire Male and Immunocastrated Pigs for Meat Products—An Overview with Recommendations. Animals.

[15] CIE (1986). Official Recommendations of the International Commission on Illumination.

[16] Honikel K.O. (1998). Reference Methods for the Assessment of Physical Characteristics of Meat. Meat Sci..

[17] Grau R., Hamm R. (1953). Eine Einfache Methode Zur Bestimmung Der Wasserbindung Im Muskel. Naturwissenschaften.

[18] Pohja N.S., Ninivaara F.P. (1957). Die Estimmung Der Wasserbindung Des Fleisches Mittels Der Konstandruckmethods. Fleischwirtschaft.

[19] (2010). Meat and Meat Products. Determination of Contents of Fat, Protein, and Water. Near Infrared Transmission (NIT) Spectrometry Using Calibration on Artificial Neural Networks (ANN).

[20] AOAC (2007). Official Methods of Analysis of the AOAC.

[21] (2000). Animal and Vegetable Fats and Oils Preparation of Methyl Esters of Fatty Acids. Polish Standard Method PN-EN ISO.

[22] Puppel K., Kuczyńska B., Nalecz-Tarwacka T., Grodzki H. (2013). Influence of Linseed Variety on Fatty Acid Profile in Cow’s Milk. J. Sci. Food Agric..

[23] Ulbricht T.L.V., Southgate D.A.T. (1991). Coronary Heart Disease: Seven Dietary Factors. Lancet.

[24] Wojtasik-Kalinowska I., Guzek D., Górska-Horczyczak E., Brodowska M., Sun D.W., Wierzbicka A. (2018). Diet with Linseed Oil and Organic Selenium Yields Low N-6/n-3 Ratio Pork Semimembranosus Meat with Unchanged Volatile Compound Profiles. Int. J. Food Sci. Technol..

[25] Górska-Horczyczak E., Wojtasik-Kalinowska I., Guzek D., Sun D.W., Wierzbicka A. (2017). Differentiation of Chill-Stored and Frozen Pork Necks Using Electronic Nose with Ultra-Fast Gas Chromatography. J. Food Process. Eng..

[26] Pauly C., Spring P., Odoherty J.V., Ampuero Kragten S., Bee G. (2009). Growth Performance, Carcass Characteristics and Meat Quality of Group-Penned Surgically Castrated, Immunocastrated (Improvac^®^) and Entire Male Pigs and Individually Penned Entire Male Pigs. Animal.

[27] Škrlep M., Poklukar K., Kress K., Vrecl M., Fazarinc G., Lukač N.B., Weiler U., Stefanski V., Čandek-Potokar M. (2020). Effect of Immunocastration and Housing Conditions on Pig Carcass and Meat Quality Traits. Transl. Anim. Sci..

[28] Gispert M., Àngels Oliver M., Velarde A., Suarez P., Pérez J., Font i Furnols M. (2010). Carcass and Meat Quality Characteristics of Immunocastrated Male, Surgically Castrated Male, Entire Male and Female Pigs. Meat Sci..

[29] dos Santos É.R., Bridi A.M., da Silva C.A., Alfieri A.A., Fritzen J.T.T., Terto D.K., Correia E.R. (2021). Gender Effects on Pork Quality and Calpain-1 and Calpastatin Gene Expression in Male Pig Muscle. Meat Sci..

[30] Aluwé M., Langendries K.C.M., Bekaert K.M., Tuyttens F.A.M., Brabander D.L.D., De Smet S., Millet S. (2013). Effect of Surgical Castration, Immunocastration and Chicory-Diet on the Meat Quality and Palatability of Boars. Meat Sci..

[31] Corino C., Rossi R., Musella M., Pastorelli G., Cannata S. (2009). Effect of Different Production Typologies on Chemical, Physical and Sensory Characteristics of Italian Commercial Pork. J. Sci. Food Agric..

[32] Ba H.V., Seo H.W., Seong P.N., Cho S.H., Kang S.M., Kim Y.S., Moon S.S., Choi Y.M., Kim J.H. (2019). Live Weights at Slaughter Significantly Affect the Meat Quality and Flavor Components of Pork Meat. Anim. Sci. J..

[33] Cheng Q., Sun D.W. (2008). Factors Affecting the Water Holding Capacity of Red Meat Products: A Review of Recent Research Advances. Crit. Rev. Food Sci. Nutr..

[34] Roy B.C., Bruce H.L. (2024). Contribution of Intramuscular Connective Tissue and Its Structural Components on Meat Tenderness-Revisited: A Review. Crit. Rev. Food Sci. Nutr..

[35] Paul C., Leser S., Oesser S. (2019). Significant Amounts of Functional Collagen Peptides Can Be Incorporated in the Diet While Maintaining Indispensable Amino Acid Balance. Nutrients.

[36] Škrlep M., Tomažin U., Lukač N.B., Poklukar K., Čandek-Potokar M. (2019). Proteomic Profiles of the Longissimus Muscles of Entire Male and Castrated Pigs as Related to Meat Quality. Animals.

[37] Latorre M.A., Lázaro R., Valencia D.G., Medel P., Mateos G.G. (2004). The Effects of Gender and Slaughter Weight on the Growth Performance, Carcass Traits, and Meat Quality Characteristics of Heavy Pigs. J. Anim. Sci..

[38] Grela E.R., Świątkiewicz M., Kowalczuk-Vasilev E., Florek M., Kosior-Korzecka U., Skałecki P. (2020). An Attempt of Implementation of Immunocastration in Swine Production—Impact on Meat Physicochemical Quality and Boar Taint Compound Concentration in the Meat of Two Native Pig Breeds. Livest. Sci..

[39] Božičković I., Savić R., Panella-Riera N., Radojković D., Brun A., Font-i-Furnols M. (2025). Pork Quality and Histological Properties of Longissimus Muscle from Boars and Early and Late Immunocastrated Pigs. Meat Sci..

[40] Krasnowska G., Salejda A.M. (2008). Wybrane Cechy Jakościowe Tłuszczu Pochodzącego z Tusz Tuczników Różnych Grup Genetycznych. Żywność Nauka-Technol.-Jakość.

[41] Salejda A., Krasnowska G., Blaszczuk M. (2009). Analysis of Quality Properties in Raw Meat and Fats from Fatteners Breeding in Wielkopolska. Acta Sci. Polonorum. Med. Vet..

[42] Olkiewicz M., Moch P. (2011). Zawartość Głównych Ilościowo Składników Surowcowych w Wybranych Elementach Zasadniczych Półtuszy Wieprzowej Oraz Ich Skład Podstawowy. Postępy Nauk. i Technol. Przemysłu Rolno-Spożywczego.

[43] Poklukar K., Čandek-Potokar M., Vrecl M., Batorek-Lukač N., Fazarinc G., Kress K., Weiler U., Stefanski V., Škrlep M. (2021). The Effect of Immunocastration on Adipose Tissue Deposition and Composition in Pigs. Animal.

[44] Kress K., Hartung J., Jasny J., Stefanski V., Weiler U. (2020). Carcass Characteristics and Primal Pork Cuts of Gilts, Boars, Immunocastrates and Barrows Using AutoFOM III Data of a Commercial Abattoir. Animals.

[45] Wood J.D., Enser M., Fisher A.V., Nute G.R., Sheard P.R., Richardson R.I., Hughes S.I., Whittington F.M. (2008). Fat Deposition, Fatty Acid Composition and Meat Quality: A Review. Meat Sci..

[46] Dinh T.T., To K.V., Schilling M.W. (2021). Fatty Acid Composition of Meat Animals as Flavor Precursors. Meat Muscle Biol..

[47] Enser M., Hallett K., Hewitt B., Fursey G.A.J., Wood J.D. (1996). Fatty Acid Content and Composition of English Beef, Lamb and Pork at Retail. Meat Sci..

[48] Skiba G., Raj S., Poławska E. (2013). Profile of Fatty Acids and Activity of Elongase and Δ5 and Δ9 Desaturase of Growing Pigs Differ in Concentration of Intramuscular Fat in Musculus Longissimus Dorsi. Anim. Sci. Pap. Rep..

[49] Gandemer G. (2002). Lipids in Muscles and Adipose Tissues, Changes during Processing and Sensory Properties of Meat Products. Meat Sci..

[50] Xu R., Molenaar A.J., Chen Z., Yuan Y. (2025). Mode and Mechanism of Action of Omega-3 and Omega-6 Unsaturated Fatty Acids in Chronic Diseases. Nutrients.

[51] Dutkowska A., Rachoń D. (2015). Role of N-3 and n-6 Unsaturated Fatty Acids in the Prevention of Cardiovascular Diseases. Chor. Serca i Naczyń.

[52] Grela E.R., Kowalczuk-Vasilev E., Klebaniuk R. (2013). Performance, Pork Quality and Fatty Acid Composition of Entire Males, Surgically Castrated or Immunocastrated Males, and Female Pigs Reared under Organic. Pol. J. Vet. Sci..

[53] Covaciu F.D., Feher I., Cristea G., Dehelean A. (2024). Nutritional Quality and Safety Assessment of Pork Meat Cuts from Romania: Fatty Acids and Elemental Profile. Foods.

[54] Metz C., Hohl K., Waidelich S., Drochner W., Claus R. (2002). Active Immunization of Boars against GnRH at an Early Age: Consequences for Testicular Function, Boar Taint Accumulation and N-Retention. Livest. Prod. Sci..

[55] Brunius C., Zamaratskaia G., Andersson K., Chen G., Norrby M., Madej A., Lundström K. (2011). Early Immunocastration of Male Pigs with Improvac^®^—Effect on Boar Taint, Hormones and Reproductive Organs. Vaccine.

[56] Pawlicki P., Galuszka A., Pardyak L., Tuz R., Płachno B.J., Malopolska M., Dubniewicz K., Yang P., Kotula-Balak M., Tarasiuk K. (2022). Leydig Cells in Immunocastrated Polish Landrace Pig Testis: Differentiation Status and Steroid Enzyme Expression Status. Int. J. Mol. Sci..

[57] Fazarinc G., Batorek-Lukač N., Škrlep M., Poklukar K., Van den Broeke A., Kress K., Labussière E., Stefanski V., Vrecl M., Čandek-Potokar M. (2023). Male Reproductive Organ Weight: Criteria for Detection of Androstenone-Positive Carcasses in Immunocastrated and Entire Male Pigs. Animals.

[58] Zeng X.Y., Turkstra J.A., Meloen R.H., Liu X.Y., Chen F.Q., Schaaper W.M.M., Oonk H.B., Guo D.Z., Van de Wiel D.F.M. (2002). Active Immunization against Gonadotrophin-Releasing Hormone in Chinese Male Pigs: Effects of Dose on Antibody Titer, Hormone Levels and Sexual Development. Anim. Reprod. Sci..

[59] Kubale V., Batorek N., Škrlep M., Prunier A., Bonneau M., Fazarinc G., Čandek-Potokar M. (2013). Steroid Hormones, Boar Taint Compounds, and Reproductive Organs in Pigs According to the Delay between Immunocastration and Slaughter. Theriogenology.

[60] Čandek-Potokar M., Prevolnik M., Škrlep M. Testes Weight Is Not a Reliable Tool for Discriminating Immunocastrates from Entire Males. Proceedings of the International Symposium on Animal Science.

[61] Van den Broeke A., Aluwé M., Kress K., Stefanski V., Škrlep M., Batorek N., Ampe B., Millet S. (2022). Effect of Dietary Energy Level in Finishing Phase on Performance, Carcass and Meat Quality in Immunocastrates and Barrows in Comparison with Gilts and Entire Male Pigs. Animal.

[62] Werner D., Baldinger L., Bussemas R., Büttner S., Weißmann F., Ciulu M., Mörlein J., Mörlein D. (2021). Early Immunocastration of Pigs: From Farming to Meat Quality. Animals.

[63] Škrlep M., Batorek N., Bonneau M., Fazarinc G., Šegula B., čandek-Potokar M. (2012). Elevated Fat Skatole Levels in Immunocastrated, Surgically Castrated and Entire Male Pigs with Acute Dysentery. Vet. J..

[64] Weiler U., Götz M., Schmidt A., Otto M., Müller S. (2013). Influence of Sex and Immunocastration on Feed Intake Behavior, Skatole and Indole Concentrations in Adipose Tissue of Pigs. Animal.

[65] Zamaratskaia G., Andersson H.K., Chen G., Andersson K., Madej A., Lundström K. (2008). Effect of a Gonadotropin-Releasing Hormone Vaccine (Improvac^TM^) on Steroid Hormones, Boar Taint Compounds and Performance in Entire Male Pigs. Reprod. Domest. Anim..

[66] Needham T., Gous R.M., Lambrechts H., Pieterse E., Hoffman L.C. (2020). Combined Effect of Dietary Protein, Ractopamine, and Immunocastration on Boar Taint Compounds, and Using Testicle Parameters as an Indicator of Success. Foods.

[67] Škrlep M., Šegula B., Zajec M., Kastelic M., Košorok S. (2010). Effect of Immunocastration (Improvac^®^) in Fattening Pigs I: Growth Performance, Reproductive Organs and Malodorous Compounds. Slov. Vet. Res..

[68] Aleksić J., Dokmanović M., Aleksić Z., Teodorović V., Stojić V., Trbović D., Baltić M.Z. (2012). Investigation of The Efficacy of Immunocastration Aimed at The Prevention of Sex Odour in Boar Meat. Acta Vet..

[69] Stupka R., Čítek J., Vehovský K., Zadinová K., Okrouhlá M., Urbanová D., Stádník L. (2017). Effects of Immunocastration on Growth Performance, Body Composition, Meat Quality, and Boar Taint. Czech J. Anim. Sci..

[70] Han X., Zhou M., Cao X., Du X., Meng F., Bu G., Kong F., Huang A., Zeng X. (2019). Mechanistic Insight into the Role of Immunocastration on Eliminating Skatole in Boars. Theriogenology.

[71] Chen G., Zamaratskaia G., Madej A., Lundström K. (2006). Effect of HCG Administration on the Relationship between Testicular Steroids and Indolic Compounds in Fat and Plasma in Entire Male Pigs. Meat Sci..

[72] Meinert L., Lund B., Bejerholm C., Aaslyng M.D. (2017). Distribution of Skatole and Androstenone in the Pig Carcass Correlated to Sensory Characteristics. Meat Sci..

[73] Rius M.A., García-Regueiro J.A. (2001). Skatole and Indole Concentrations in Longissimus Dorsi and Fat Samples of Pigs. Meat Sci..

[74] Thomsen R., Edwards S.A., Jensen B.B., Rousing T., Sorensen J.T. (2015). Effect of Faecal Soiling on Skatole and Androstenone Occurrence in Organic Entire Male Pigs. Animal.

[75] Kjeldsen N. (1993). Practical Experience with Production and Slaughter of Entire Male Pigs.

[76] Czech A., Kusior G., Zięba G., Łukaszewicz M., Grela E.R. (2022). Effect of Immunocastration on the Steroid Hormones Content, Serum Lipid Profile, and Fatty Acid Profile in Tissues of Porkers Fed Dry or Wet Diet. Anim. Sci. Pap. Rep..

[77] Tuz R. (2008). Zapobieganie Wystepowaniu Zapachu Plciowego w Tuszach Niekastrowanych Chirurgicznie Knurkow. Zesz. Nauk. Akad. Rol. w Krakowie. Rozpr..

